# Frequency of change determines effectiveness of microbial response strategies

**DOI:** 10.1038/s41396-023-01515-9

**Published:** 2023-09-18

**Authors:** Shengjie Li, Damon Mosier, Xiaoli Dong, Angela Kouris, Guodong Ji, Marc Strous, Muhe Diao

**Affiliations:** 1 Department of Geoscience, University of Calgary, Calgary, AB T2N 1N4 Canada; 2 Key Laboratory of Water and Sediment Sciences, Ministry of Education, Department of Environmental Engineering, Peking University, 100871 Beijing, China; 3 Department of Biogeochemistry, Max Planck Institute for Marine Microbiology, 28359 Bremen, Germany

**Keywords:** Microbial ecology, Microbial ecology

## Abstract

Nature challenges microbes with change at different frequencies and demands an effective response for survival. Here, we used controlled laboratory experiments to investigate the effectiveness of different response strategies, such as post-translational modification, transcriptional regulation, and specialized versus adaptable metabolisms. For this, we inoculated replicated chemostats with an enrichment culture obtained from sulfidic stream microbiomes 16 weeks prior. The chemostats were submitted to alternatingly oxic and anoxic conditions at three frequencies, with periods of 1, 4 and 16 days. The microbial response was recorded with 16S rRNA gene amplicon sequencing, shotgun metagenomics, transcriptomics and proteomics. Metagenomics resolved provisional genomes of all abundant bacterial populations, mainly affiliated with Proteobacteria and Bacteroidetes. Almost all these populations maintained a steady growth rate under both redox conditions at all three frequencies of change. Our results supported three conclusions: (1) Oscillating oxic/anoxic conditions selected for generalistic species, rather than species specializing in only a single condition. (2) A high frequency of change selected for strong codon usage bias. (3) Alignment of transcriptomes and proteomes required multiple generations and was dependent on a low frequency of change.

## Introduction

“The only constant in life is change”, according to the philosopher Heraclitus, ~500 BC in Ancient Greece. “Changes are in diverse forms, up or down, rigid or flexible, and throughout the whole universe”, as stated in I Ching, an ancient Chinese divination text, ~1000 BC. Microbes, the smallest and most abundant cellular organisms, are coping with change all the time. Microbiomes of the oral and digestive-tract of animals experience dynamics associated with feeding regimes, leading to cycles of feast and famine multiple times per day [1, 2]. Cyanobacteria display a progression of gene expression in response to diurnal cycles [3, 4]. Seasons dictate change in lakes, with water columns mixing in winter and stratifying during summer [5]. Often, change affects redox conditions and triggers microbial response, which is the topic of this study.

Even though it is often assumed that microbes are always responsive to their environment, this is not necessarily the case. After all, regulation is associated with trade-offs, such as the bio-energetic costs associated with accelerated turnover of the proteome. Both protein biosynthesis and protein degradation cost energy and consume ATP [6]. Instead of responding to change, microbes may survive a period of unfavorable conditions without adaptation, counting on conditions to become more favorable quickly enough. Alternatively, they may constitutively express a multifunctional proteome that provides answers to different conditions [7]. For example, in bioreactors cycled every 6–12 h, relatively few proteins were found responsive between oxic and anoxic phases [8, 9]. In intertidal sediments, transcription of genes for aerobic respiration and denitrification was not affected by oxygen concentrations [10]. In tropical forest soils, many taxa displayed sustained activity through rapidly fluctuating redox conditions [11].

Acclimatization can also occur on the community level. Generalists with multiple physiological capabilities are able to deal with broader redox regimes than specialists focusing on a single metabolism [12].

Here, we investigate to what extent the frequency of change favors—selects for—specific metabolic and ecological strategies and adaptations, such as (1) generalism versus specialism, (2) use of post-translational modifications versus transcriptional regulation.

**Fig. 1:**
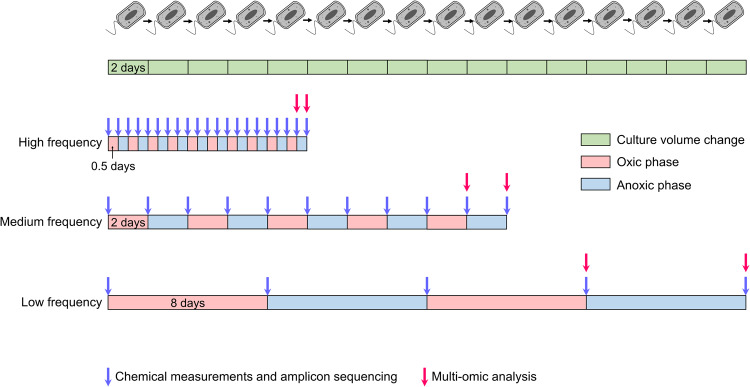
Experimental design of this study. Alternating phases of oxic and anoxic conditions were established in the three sets of triplicated chemostats. The phases differed in length for each set, but the dilution rate was the same for all chemostats.

When a wild microbiome is first transferred to the lab, initial selection may be governed by factors outside the scope of a study’s design—for example, the growth medium may be toxic to some of the microbes present in the natural sample. On the other hand, when an enrichment proceeds for too long, evolutionary adaptation to the experimental setup may become a confounding factor [13, 14]. To strike a balance between these two, we used a 16-week pre-adaptation period in batch-incubations, followed by the actual experiments conducted in chemostats, for at most 16 generations, at a dilution rate of 0.5 volume changes per day. This corresponds to a doubling time of 1.4 days, much longer than typical for isolated bacteria grown in the lab but in the range of doubling times of most wild microbes [15]. We applied three different change regimes (Fig. 1). In the first set of triplicated chemostats, cells experienced alternatingly oxic and anoxic conditions about twice per generation. In the second set, redox conditions changed in pace with generation time. In the third set, the cells experienced redox change about once per four generations. We monitored microbial responses by transcriptomics and proteomics.

## Materials and methods

### Sampling and pre-incubation

Sediment samples were collected at six sampling sites from sulfidic streams at Canyon Creek, Canada (50.95159°N, 114.55951°W) on March 5th, 2020 (Fig. S1). In total, 60 g of the mixed sediments were inoculated into six 1 l serum bottles with 600 ml fresh medium. The medium contained MgCl_2_ • 6H_2_O (2 mM), KH_2_PO_4_ (0.7 mM), CaCl_2_ (0.9 mM), NH_4_Cl (1.9 mM), Na_2_SO_4_ (2.5 mM), NaNO_3_ (1 mM), sodium acetate (2 mM), FeCl_2_ (10 mM), NaHCO_3_ (20 mM), trace element solution (1 ml/l) and vitamin solution (1 ml/l). Trace element solution contained (per liter) titriplex III (EDTA) (0.5 g), FeSO_4_ • 7H_2_O (0.2 g), ZnSO_4_ • 7H_2_O (0.01 g), MnCl_2_ • 4H_2_O (0.003 g), H_3_BO_3_ (0.03 g), CoCl_2_ • 6H_2_O (0.02 g), CuCl_2_ • 2H_2_O (0.001 g), NiCl_2_ • 6H_2_O (0.002 g) and Na_2_MoO_4_ • 2H_2_O (0.003 g). Vitamin solution contained (per liter) biotin (0.1 g), 4-aminobenzoic acid (0.5 g), calcium pantothenate (0.1 g), thiamin (0.2 g), nicotinic acid (1.0 g), pyridoxamine (2.5 g) and vitamin B12 (0.1 g). The six serum bottles were incubated in the dark, at room temperature in a shaker for 16 weeks, for lab acclimatization. During acclimatization, the bottles were alternately incubated with and without oxygen for 1 week (8 oxic phases, 8 anoxic phases). At the beginning of each oxic phase, the bottles were flushed with helium. Then, oxygen was injected into each bottle with a syringe and the final concentration was about 7%. At the beginning of each anoxic phase, the bottles were only flushed with helium. Sodium acetate (2 mM), NaNO_3_ (1 mM) and NaHCO_3_ (20 mM) (final concentrations) were added to the bottles every 4 weeks.

### Chemostat incubation

After 16 weeks, the acclimatized cultures were used to inoculate chemostat incubations. For this, the six cultures were first mixed together and then used as inoculum for three sets of triplicated chemostats. In total, 100 ml of mixed acclimatized culture were added to each 1 l chemostat with 900 ml fresh medium. The fresh medium contained MgCl_2_ • 6H_2_O (2 mM), KH_2_PO_4_ (0.7 mM), CaCl_2_ (0.9 mM), NH_4_Cl (1.9 mM), Na_2_SO_4_ (5 mM), NaNO_3_ (2 mM), sodium acetate (5 mM), NaHCO_3_ (20 mM), trace element solution (1 ml/l) and vitamin solution (1 ml/l) and L-cysteine (5 mM). L-cysteine solution was filter sterilized in an anaerobic chamber and kept anoxic before it was added to the medium bottles. The final pH was 6.5–7.5.

Each chemostat setup consisted of a 1 l medium (feed) bottle, a 1 l magnetically stirred culture bottle and an effluent collection bottle (Fig. S2). Fresh medium was pumped from the medium bottle to the culture bottle at a rate of 0.5 l per day (one volume change per 2 days). The total culture volume of the culture bottles was maintained at 1 l by pumping out the excess culture volume to the effluent collection bottle. All culture bottles of the chemostats were covered with aluminum foil and stirred at 300 rounds per minute with a magnetic stir bar. During oxic phases, 10 ml/min air was supplied to the medium bottle and the culture bottle. During anoxic phases, 10 ml/min Argon was supplied to the medium bottle and the culture bottle. Thus, the chemostats experienced alternatingly oxic and anoxic conditions. Two mM FeCl_2_ was added directly to the culture bottles at the beginning of every oxic phase. Chemostats were started with an oxic phase and ended with an anoxic phase.

There were three treatments for the chemostat incubations, high-frequency, medium-frequency and low-frequency (Fig. 1). For each treatment, a set of triplicated chemostats was run. The nine chemostats were operated independently in parallel with the same inoculum. For high-frequency experiments, each phase lasted for 0.5 days and the total culture time was 10 days (10 oxic phases and 10 anoxic phases, 5 culture volume changes). For medium-frequency experiments, each phase lasted for 2 days and the total culture time was 20 days (5 oxic phases and 5 anoxic phases, 10 culture volume changes). For low-frequency experiments, each phase lasted for 8 days and the total culture time was 32 days (2 oxic phases and 2 anoxic phases, 16 culture volume changes).

Culture samples were collected immediately at the beginning of the incubations and at the end of every phase. The samples were centrifuged at 5000 rpm for 10 min. Subsequently, 0.2 μm-filtered supernatants were used for chemical measurements and cell pellets were used for DNA, RNA and protein extractions.

### Chemical measurements

Sulfide, ammonia, ferrous iron and nitrite were determined with an Evolution 260 Bio UV-Visible Spectrophotometer (Thermo Scientific, CA, USA). Ammonia was measured by the indophenol reaction [16]. Nitrite was measured by a reaction with sulfanilamide and N-(1-naphthyl)ethylenediamine [17]. Sulfide was fixed with zinc acetate and determined by a reaction with dimethylparaphenylenediamine and Fe(NH_4_)(SO_4_)_2_ • 12 H_2_O as previously described [18]. Ferrous iron was measured with the ferrozine method [19]. Acetate was quantified by high-performance liquid chromatography (HPLC) using a Thermo RS3000 HPLC fitted with a Kinetex 2.6 μm EVO C18 100 Å LC column, a Thermo RS3000 pump and an UltiMate 3000 fluorescence detector (Thermo Scientific, CA, USA). Nitrate and sulfate were measured by a Dionex ICS-5000 Ion Chromatography System (Thermo Scientific, CA, USA) equipped with an anion-exchange column (Dionex IonPac AS22; 4 × 250 mm; Thermo Scientific), an EGC-500 K_2_CO3 eluent generator cartridge and a conductivity detector.

### DNA extraction and amplicon sequencing

DNA was extracted from sediments, pre-incubated culture pellets and chemostat culture pellets with the FastDNA SPIN Kit for Soil (MP Biomedicals, Solon, OH, USA). Qubit 2.0 Fluorometer (Invitrogen, CA) was used to quantify the DNA concentration. Amplicon sequencing was performed with the primers A519F (5’-CAGCMGCCGCGGTAA-3’) and Pro805R (5’-GACTACNVGGGTATCTAATCC-3’), targeting both archaea and bacteria [20, 21]. PCR systems were prepared with template DNA, the forward and the reverse primers and 2x KAPA HiFi Hot Start Ready Mix (Roche, CA). PCR was performed with the following protocol: an initial denaturation cycle (95 °C for 3 min), 25 cycles of denaturation (95 °C for 30 s), annealing (55 °C for 45 s) and extension (72 °C for 60 s), and a final extension cycle (72 °C for 5 min). Triplicated PCR reactions were conducted for each DNA sample and the PCR products were verified by 1% agarose gel electrophoresis. The amplicons were pooled, purified and sequenced with a Miseq System (Illumina, San Diego, CA) using the 2 × 300 bp MiSeq Reagent Kit v3. Raw data was processed with amplicon sequencing variant (ASV) analysis in MetaAmp [22]. Non-metric multidimensional scaling (NMDS) analysis was performed with the “vegan” package in R v4.0.3. Different groups were labeled with the “ordiellipse” function, which invisibly returns an object that has a summary method that returns the coordinates of centroids and areas of ellipses. A total of 123 samples collected from the sediments and cultures were sequenced, yielding 4,632,919 reads after quality control (4858 to 169,903 reads per sample).

### Metagenomic sequencing and data analysis

Eighteen samples were selected for metagenomic, metatranscriptomic and metaproteomic analysis. These samples were collected at the final oxic and anoxic phases of the triplicated treatments (2 × 3 × 3 = 18). For metagenomics, extracted DNA (see above) was fragmented to an average insert size of ~350 bp using acoustic sonication (Covaris model S220). Adapter-ligated fragment libraries were generated using the Kapa Biosystems HyperPrep PCR-free library preparation workflow, according to the manufacturer’s protocol. The libraries were quantified with the KAPA qPCR library quantitation assay and sequenced on a NextSeq 500 system (Illumina, San Diego, CA) with the 300 cycle Mid-Output Kit (2 × 151 bp paired end sequencing). The final output was ~7.2 M read pairs (~2.2 Gb) per sample.

Raw reads were filtered with BBduk. First, the last base off of 151 bp reads was trimmed with “ftm = 5”. Adapters were clipped off with “tbo tpe k = 23 mink = 11 hdist = 1 ktrim = r”. PhiX sequences were filtered out with “k = 31 hdist = 1”. 3’ low quality bases were clipped off with “qtrim=rl trimq=15 minlength=30”. Quality-controlled reads were assembled separately for each sample and co-assembled for all samples with MEGAHIT v1.2.2-beta [23]. Contigs shorter than 500 bp were not considered for further analysis. Per contig sequencing depth was determined with BBMap v38.06 with the parameter “minid=0.99”. The coassembly and each individually assembled sample were binned separately by three methods, MetaBat v2:2.15 [24], Maxbin v2.2.7 [25] and CONCOCT v1.1.0 [26]. DASTOOL v1.1.2 was applied to select the best bins from the three binning methods for each library [27]. dRep v3.0.0 was used to dereplicate bins obtained from different assemblies [28]. Completeness and contamination of bins (MAGs, Metagenome-Assembled-Genomes) were estimated by CheckM v1.1.3 [29]. MAGs were taxonomically classified with GTDBtk v1.3.0 [30]. Unbinned contigs were dereplicated by blast searches to each other. If an unbinned contig was 99% identical to a binned contig, the unbinned contig was discarded. If two unbinned contigs had 99% identity to each other, only the longer one was kept. Sequencing depth information of all non-redundant contigs were aggregated from mapping results using the MetaBAT’s “jgi_summarize_bam_contig_depths” script [24]. Contigs were annotated using MetaErg [31].

The relative sequence abundance of each population associated with a MAG in metagenomes was calculated by dividing the sequencing depth of the MAG by the sum of sequencing depths of all MAGs and the unbinned contigs. Each MAG was associated with corresponding ASVs based on abundance and taxonomy. The replication rate of each population was estimated with iRep [32].

### RNA extraction and metatranscriptomic sequencing

Pellets from 50 ml culture were processed for RNA extraction using the RNeasy PowerSoil Total RNA Kit (Qiagen, USA). A DNase kit (Invitrogen, CA) was used for RNA purification. The RNA concentration was quantified with a Qubit 2.0 Fluorometer (Invitrogen, CA). Libraries were prepared using the New England Biolabs NEBNext rRNA depletion kit (Bacteria) and NEBNext Ultra II Directional RNA library prep kit (Illumina, San Diego, CA). The libraries were quantified by KAPA qPCR library quantitation assays and sequenced paired-end using the MiSeq system (Illumina, San Diego, CA) with a 150 cycle v3 sequencing kit, yielding ~1.5 M reads pairs for each sample.

Read quality control was performed using the procedure described above. Reads mapping to ribosomal genes were filtered out with SortMeRNA v4.2.0 with a 1 × e^−10^ e-value cutoff [33]. The filtered reads were mapped to the dereplicated contigs with 99% identity. Relative transcriptional activity for each gene was calculated based on per base sequencing depth.

To investigate transcriptional regulation of each gene in each MAG, transcriptional abundance of each gene in each MAG was normalized by total transcriptional abundance of all genes in the MAG. Relative abundance of transcripts dedicated to key metabolic processes in each MAG was calculated. Transcriptome turnover of each MAG was defined as the percentage of gene transcripts that were different between two phases or replicates. For transcriptome turnover calculations, only genes with sequencing depths of ≥20 in the sum of two phases or replicates were included. Transcriptome turnover for each MAG was calculated by dividing the sum of the absolute differences in normalized transcriptome sequencing depth of the genes in the MAG by two and by the number of included genes. Transcriptome turnover was compared to expected growth of a population, assuming per population abundances did not change between phases (Supplementary Method). If the transcriptome turnover was higher than the theoretical no-change value, this was taken as evidence that a population was actively degrading old transcripts.

### Protein extraction and metaproteomics

For protein extraction, 50 ml culture pellets were transferred to lysing matrix bead tubes A (MP Biomedicals) with the addition of SDT-lysis buffer (0.1 M DTT) in a 10:1 ratio [34]. Matrix tubes were bead-beated in an OMNI Bead Ruptor 24 for 45 s at 6 m s^−1^ and then incubated at 95 °C for 10 min. These steps led to pelleted, lysed cells. Peptides were isolated from pellets by filter-aided sample preparation (FASP) [35]. A Qubit 2.0 Fluorometer (Invitrogen, CA) was used to quantify protein concentrations. For proteomics, peptides were first separated on a 50 cm × 75 μm analytical EASY-Spray column by an UltiMate 3000 RSLCnano Liquid Chromatograph (Thermo Fisher Scientific, Waltham, MA, USA) as previously described [36]. Eluting peptides were analyzed in a QExactive Plus hybrid quadrupole-Orbitrap mass spectrometer (Thermo Fisher Scientific, CA, USA).

Expressed proteins were identified and quantified with Proteome Discoverer version 2.0.0.802 (Thermo Fisher Scientific, CA, USA), using the Sequest HT node [34]. The Percolator Node and FidoCT were used to estimate false discovery rates (FDR) at the peptide and protein level, respectively. Peptides and proteins with FDR > 5% were discarded [36]. Relative abundance of proteins was estimated based on normalized peptide-spectral matches (PSMs). The identification database was prepared based on predicted protein sequences of all binned and unbinned contigs. Redundant proteins (>95% amino acid identity) were removed by cd-hit [37], while giving preference to proteins from binned contigs [34]. Phosphorylated and acetylated proteins were identified in parallel. Only unambiguous PSMs with “high” FDR confidence were included in further phosphorylation and acetylation analysis. In total, 2,216,073 MS/MS spectra were acquired, yielding 616,029 PSMs, 20,928 identified proteins and 10,655 proteins of at least “medium” confidence.

Proteomic abundance of each gene in each MAG was normalized by total protein abundance of all genes in the MAG. Relative abundance of proteins dedicated to key metabolic processes in each MAG was calculated. Proteome turnover of each MAG was defined as the percentage of proteins that were different between two phases or replicates. For proteome turnover calculations, only genes with ≥10 detected PSMs in the sum of two phases or replicates were included. Proteome turnover for each MAG was calculated by dividing the sum of the absolute relative protein abundance differences of the genes in the MAG by two and by the number of included genes. Proteome turnover was compared to expected growth of a population, assuming per population abundances did not change between phases (Supplementary Method). If the proteome turnover was higher than the theoretical no-change value, this was taken as evidence that a population was actively degrading old proteins. Calculation of correlation between transcriptional and translational regulation only included genes with the sum of transcript sequencing depths ≥60 and the sum of detected PSMs ≥30 in triplicates in two phases. For each MAG, the Pearson correlation coefficient between the transcriptome differences and the proteome differences of the involved genes between phases was calculated.

### Statistical analyses

Calculations of transcriptome/proteome turnover and their correlation were performed with the “tidyverse” package in R v4.0.3. Nutrient concentrations, DNA concentrations and relative abundance of microbial populations were compared between phases and between frequencies in *t*-tests for independent samples. For the comparison between phases, *t*-tests were performed between all samples collected at the oxic phases and all samples collected at the anoxic phases. iRep values were compared between phases in *t*-tests for paired samples. Fold changes in activity of genes associated with key metabolic subsystems were compared to 1 in *t*-test for one sample. Transcriptome/proteome turnover and correlation values of single populations were compared within and between frequencies in *t*-tests for independent samples. Transcriptome/proteome turnovers of single populations were compared to theoretical cell turnovers in *t*-test for one sample. All *t*-tests were carried out in PASW Statistics v18.0. Statistical analyses were considered significant with *p* values < 0.05.

## Results

### Outcomes of microbial metabolism

**Fig. 2:**
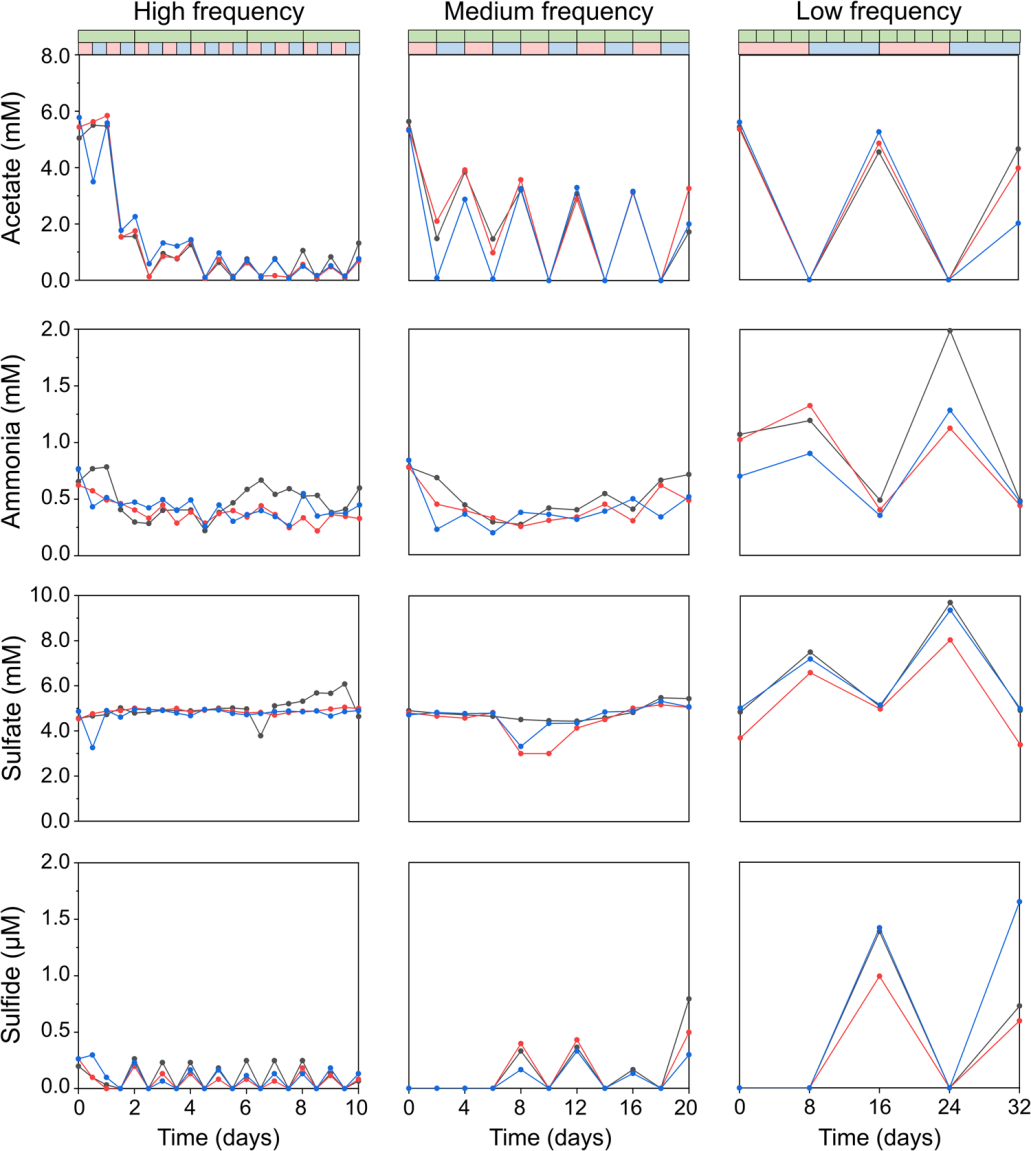
Concentrations of acetate, ammonia, sulfate and sulfide during oxic and anoxic phases at different frequencies. Triplicates are indicated by black, red and blue lines and symbols. The green bar at the top shows chemostat dilutions. In the second bar, oxic and anoxic phases are shown in red and blue respectively.

Rhythms in nutrient concentrations proceeded in pace with shifts in air and Argon flushing (Fig. 2). This showed that (1) the shifts in redox conditions were established and (2) the enriched bacteria were responding to the shifting conditions. After 2–4 generations, acetate was always fully consumed during oxic phases. During anoxic phases, it accumulated to 4.2 mM (SD = 1.1 mM) in low-frequency experiments. We observed little change in sulfate concentrations at high- (mean = 4.9 mM, SD = 0.35 mM) and medium-frequency (mean = 4.6 mM, SD = 0.57 mM). At low-frequency, sulfate accumulated to 8.1 mM (SD = 1.1 mM) during oxic phases, significantly higher than that during anoxic phases (mean = 4.8 mM, SD = 0.61 mM, two-sample two-sided *t*-tests; *p* = 0.00018). Sulfide remained undetectable during oxic phases, and accumulated to 1.1 mM (SD = 0.39 mM) during anoxic phases at low-frequency. Nitrate was mostly used up during both oxic and anoxic phases (Supplementary Table 2). These results indicated occurrence of aerobic respiration, (aerobic) denitrification, sulfide oxidation and cysteine metabolism. At low-frequency, total cellular biomass was 3.9 times (SD = 1.5) higher during oxic phases than anoxic phases (two-sample two-sided *t*-tests; *p* = 0.00026) (with DNA as a proxy for biomass, Supplementary Table 2). At medium-frequency, oxic biomass was 2.6 times (SD = 1.7) higher than anoxic biomass (two-sample two-sided *t*-tests; *p* = 0.0017). No significant differences between oxic and anoxic biomass were observed at high-frequency (two-sample two-sided *t*-tests; *p* = 0.11). Because aerobic metabolism provides more energy, biomass was expected to be higher at the end of oxic phases than anoxic phases. In addition, cells growing slower than a chemostat’s dilution rate may also be washed out during anoxic periods, reducing biomass.

### Community dynamics

**Fig. 3:**
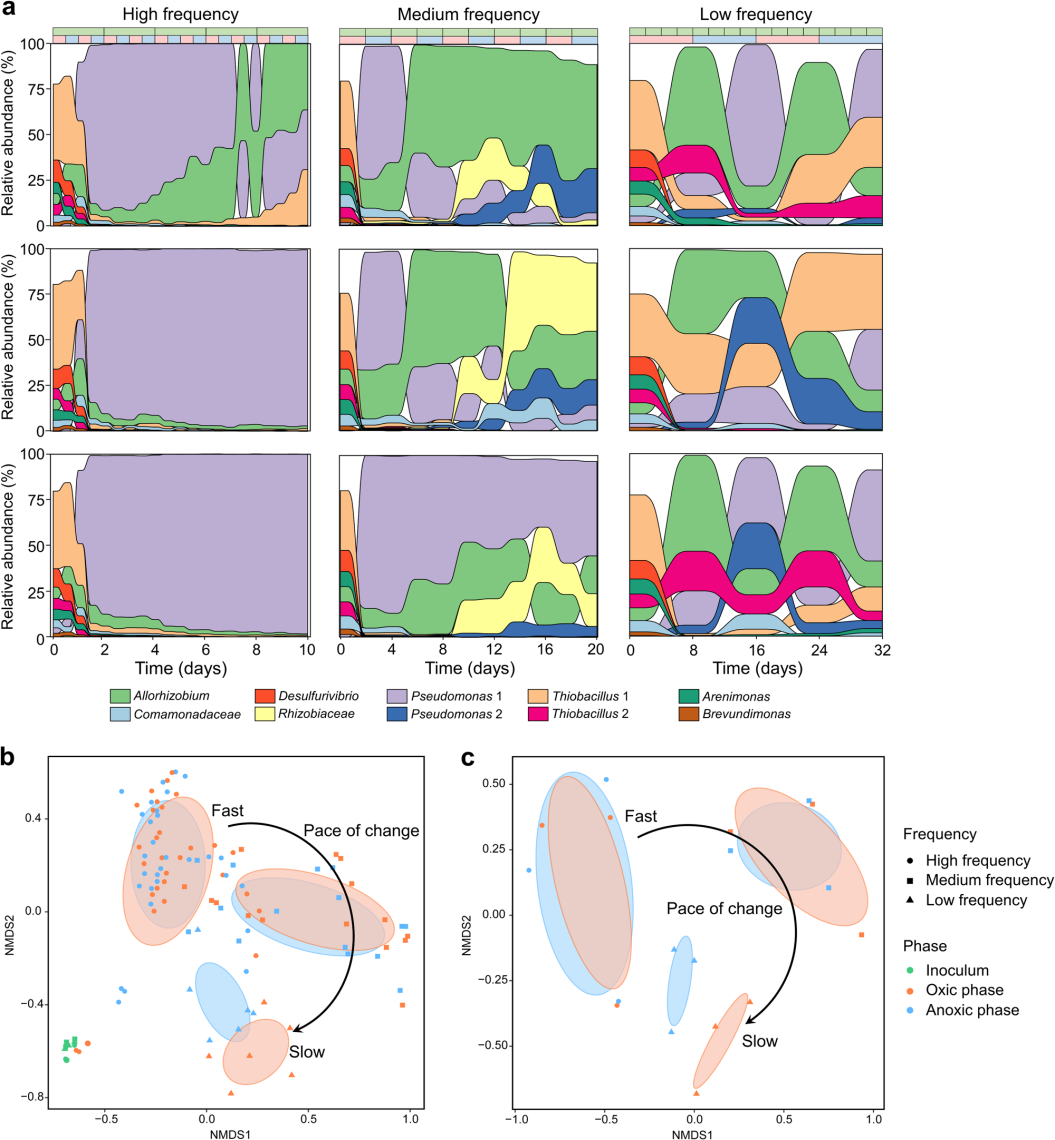
Community dynamics in the chemostats. **a** Change in relative sequence abundances of the ten most abundant populations (amplicon sequence variants, ASVs) in chemostat incubations, based on 16S rRNA gene amplicon sequencing. Outcomes of triplicated experiments are shown individually for each frequency. The green bar at the top enumerates chemostat dilutions. In the second bar, oxic and anoxic phases are shown in red and blue respectively. **b** Non-metric multidimensional scaling (NMDS) (based on Bray–Curtis distances) of all samples collected along the chemostat incubations and (**c**) only samples collected during the final oxic and the final anoxic phases. Colored ellipses show variation among samples of the two phases of each treatment obtained with the “ordiellipse” function from the “vegan” package in R.

16S rRNA gene amplicon sequencing showed both aerobes (e.g., *Thiobacillus*, *Thiothrix* and *Thiomicrorhabdus*) and anaerobes (e.g., *Geobacter*, *Desulfocapsa* and *Sulfurovum*) were present in the original sediment community (Fig. S5 and Supplementary Table 3). By the end of the 16-week pre-adaptation, 199 populations remained, with 34 of the 119 most abundant stream populations (relative sequence abundance in situ >0.1%) still present (Supplementary Table 3). This biodiversity was the starting point for subsequent selection in the main chemostat experiment. Selection in chemostats was very effective, as within 2 days, most of the 199 populations present in the inoculum became undetectable and a simple microbial community was established in each experiment. These communities featured the same ten abundant populations (ASVs), making up >88% of relative sequence abundance across all chemostat experiments (Fig. 3a and Supplementary Table 3). Three of these ten populations, “ASV **3** *Thiobacillus*”, “ASV **9** *Arenimonas*” and “ASV **10** *Brevundimonas*” were ubiquitous in the natural stream microbiomes. Among the ten most abundant populations from the stream, three were represented in the chemostats, including “ASV **3** *Thiobacillus*” (Fig. S5).

At high-frequency, we observed little turnover of populations between oxic and anoxic phases, with “ASV **1** *Pseudomonas*” dominating communities in two of the replicates. At low-frequency, community compositions oscillated in tune with oxic and anoxic conditions. For example, “ASV **1** *Pseudomonas*” (two-sample two-sided *t*-tests; *p* = 0.0037) and “ASV **2** *Allorhizobium*” (two-sample two-sided *t*-tests; *p* = 0.000072) were significantly more abundant during anoxic and oxic phases respectively. This was the only treatment where community differences between phases were larger than between replicates, as shown by NMDS (Fig. 3b, c).

### Physiology and growth of enriched populations

**Fig. 4:**
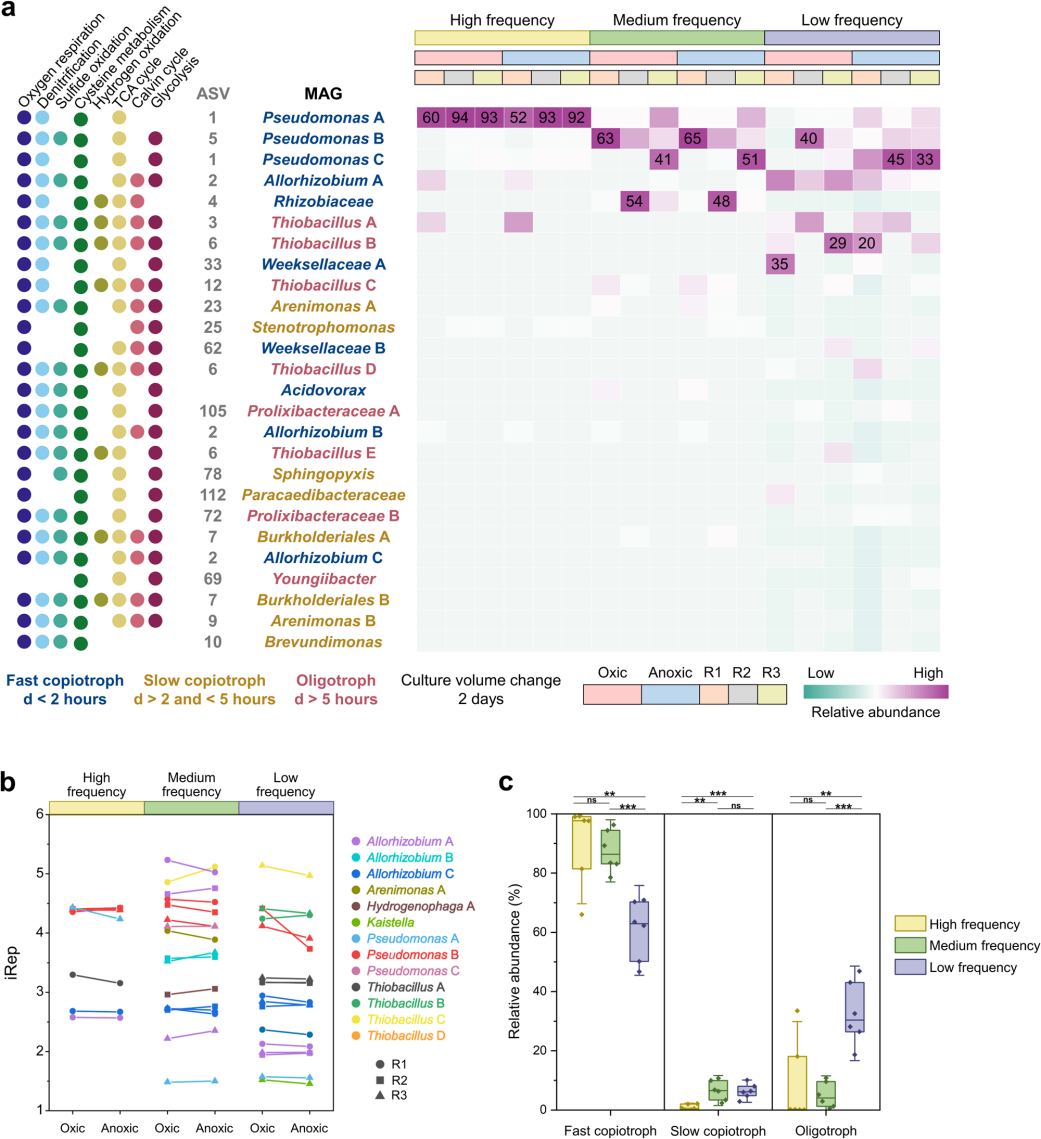
Enriched populations associated with metagenome-assembled genomes (MAGs) in the three sets of chemostats during the final oxic phase and the final anoxic phase. **a** Metabolic potential, taxonomy and relative sequence abundance of the populations (Supplementary Tables 5–31). R1, R2 and R3 represent the chemostat triplicates. Fast copiotrophs, slow copiotrophs and oligotrophs are indicated by blue, yellow and pink taxon names respectively. “d” represents the minimum doubling time predicted by gRodon [40] (Supplementary Table 33). **b** iRep values (Supplementary Table 32) of the populations. **c** Relative abundance of fast copiotrophs, slow copiotrophs and oligotrophs in the three sets of chemostats. Horizontal lines show significant differences determined in two-sample two-sided *t*-tests, with *p* values < 0.01 indicated with “**” and <0.001 indicated with “***”.

To investigate the metabolic potential and lifestyle of the enriched populations more closely, shotgun metagenomes were sequenced for samples collected at the end of the final oxic and anoxic phases of each chemostat experiment. The metagenomes were assembled and binned into metagenome-assembled genomes (MAGs). Twenty-six MAGs accounted for over 99% of sequenced DNA in all samples (Fig. 4a and Supplementary Table 5). Community composition based on 16S rRNA gene and shotgun sequencing were consistent, but relationships between ASVs and MAGs were not always one to one. For example, “ASV **1** *Pseudomonas*” was associated with two MAGs, “ASV *Pseudomonas* A” and “ASV *Pseudomonas* C”.

Analysis of gene content of MAGs indicated that 22 out of 26 associated populations were capable of both aerobic and anaerobic growth (Fig. 4a and Supplementary Tables 6–31). Most deciphered metabolic pathways were encoded in >50% of MAGs, indicating vast functional redundancy among community members. As cysteine was a major source of energy, carbon and sulfur in the medium, it was not surprising that all selected populations encoded cysteine desulfurase in their genomes. Aerobic respiration, denitrification and sulfide oxidation were common (Fig. 2). One metabolic pathway we did not expect was the Calvin cycle for carbon fixation: ten genomes contained both ribulose-1,5-bisphosphate carboxylase-oxygenase (RuBisCO) and phosphoribulokinase (PRK) [38]. Apparently, almost half of the enriched populations potentially used carbon dioxide as a carbon source even though organic substrates such as acetate were often present in excess (Fig. 2).

The capacity of most populations to grow both aerobically and anaerobically was supported by similar relative sequence abundance of associated MAGs during oxic and anoxic phases at high- and medium-frequency, in agreement with amplicon sequencing results. However, at low-frequency, “**1** *Pseudomonas* AC” and “**2** *Allorhizobium* A” were more abundant during anoxic and oxic phases respectively. To compare aerobic and anaerobic growth rates of individual populations, we calculated the peak-to-trough (PTR) ratio of sequencing depth for each MAG during oxic and anoxic conditions with iRep [32]. A high growth rate is associated with a high genome replication rate, resulting in a high PTR ratio. Although PTR is a poor proxy for growth rate when comparing different species [39], it works well for comparing growth rates of the same species across different samples [32]. We found PTR ratios did not differ significantly between phases, even for “**2** *Allorhizobium* A” (two-sample two-sided *t*-tests; *p* = 0.94) at low-frequency (Fig. 4b and Supplementary Table 32). This indicated that aerobic and anaerobic growth rates were similar. Overall, analysis of PTR ratios supported the conclusion that dynamic conditions selected for species that coped well with both oxic and anoxic conditions. Even in the case of “**1** *Pseudomonas* AC” and “**2** *Allorhizobium* A” at low-frequency, the observed changes in abundance could be explained with only minor differences in growth rate.

Differences in relative abundances of MAGs showed that frequency of change selected for specific populations, in agreement with amplicon sequencing results. For example, “**1** *Pseudomonas* AC”, “**4** *Allorhizobium* B” and “**3,6** *Thiobacillus* AB” were most abundant at high-, medium- and low-frequency respectively. To explore the potential underlying mechanisms, we investigated the codon usage bias of the MAGs with gRodon (Supplementary Table 33) [40]. Strong codon usage is associated with rapid growth, but could also facilitate rapid gene expression in response to environmental cues. According to the predicted minimum doubling times by gRodon, we classified the populations to fast copiotrophs (<2 h), slow copiotrophs (>2 and <5 h) and oligotrophs (>5 h) (Fig. 4c and Supplementary Table 33). Even though doubling times in the chemostats were the same in all experiments, they selected for a mix of copiotrophs and oligotrophs (Fig. 4c). However, fast copiotrophs were more abundant at high- and medium-frequency than at low-frequency (two-sample two-sided *t*-tests; *p* = 0.000053). Slow copiotrophs were more abundant at medium- and low-frequency (two-sample two-sided *t*-tests; *p* = 0.00028), and oligotrophs were more abundant at low-frequency (two-sample two-sided *t*-tests; *p* = 0.00013) (Fig. 4c). To generalize this finding in the natural environment, the codon usage bias of microbes experiencing redox dynamics in permeable sandy sediments was investigated [41]. The relative abundance of copiotrophs was higher at shallower sediments which experienced more frequent redox change, and decreased with depth at all three sampling sites (Supplementary Table 34). Thus, in the natural environment, strong codon usage may also be an important factor in adapting to a high pace of change.

### Transcriptional and translational regulation

**Fig. 5:**
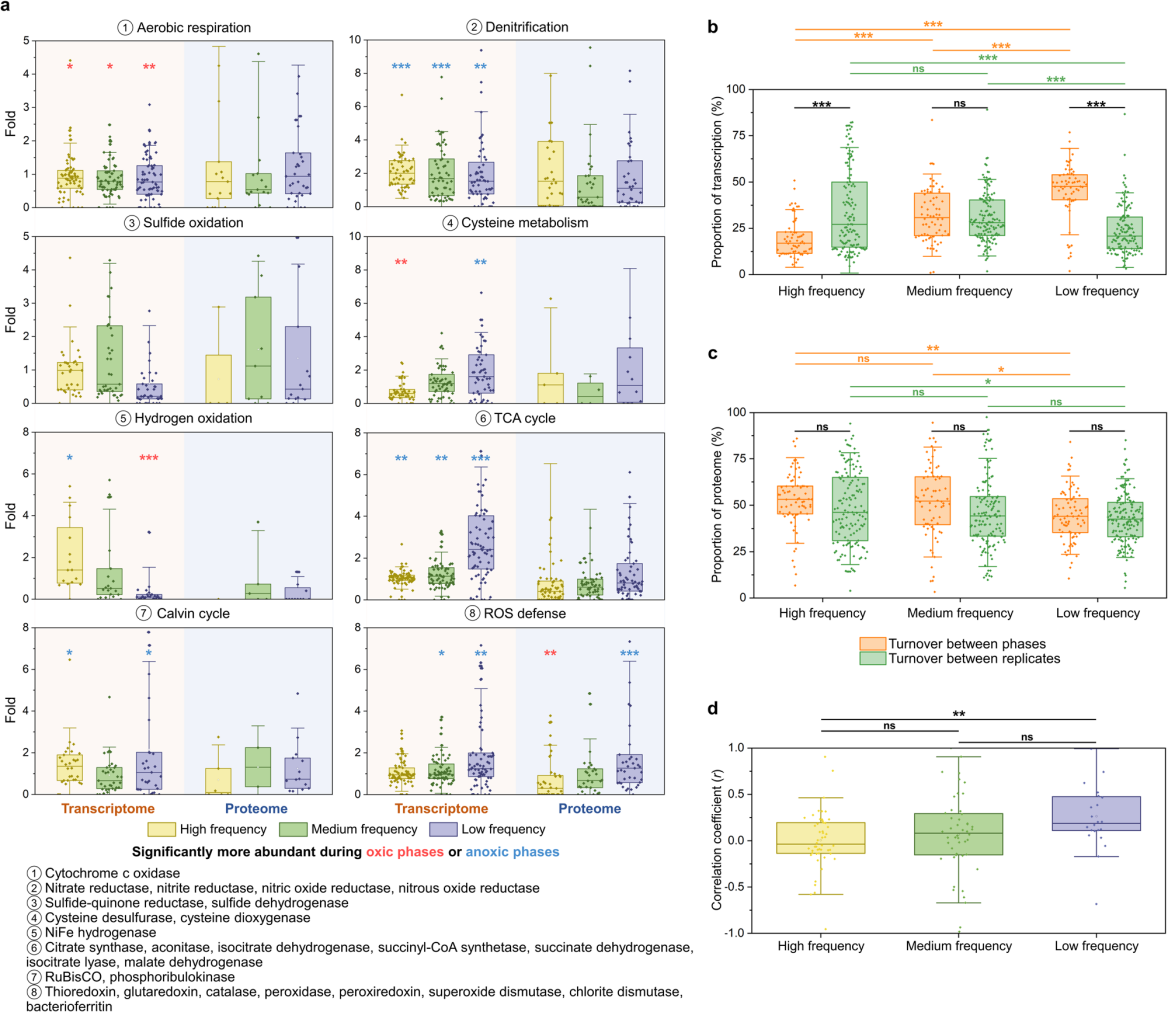
Change in transcriptomes and proteomes in the three sets of chemostats. **a** Fold change in activity of genes associated with key metabolic subsystems (Supplementary Tables 35–42) from the final oxic phase to the final anoxic phase. Each data point is associated with one of 26 MAGs. Results are shown for transcriptomes (left) and proteomes (right), each at the three different frequencies of change. Significances, determined with one-sample two-sided *t*-tests, are indicated, with *p* values < 0.05 as “*”, <0.01 as “**” and <0.001 as “***”. The enzymes involved in the analysis of each metabolism were indicated below the figure. **b** Turnover of transcriptomes across phases and replicates (Supplementary Table 43). Each dot shows overall transcriptome turnover between phases and replicates for a single MAG. **c** Turnover of proteomes across phases and replicates (Supplementary Table 43). Each dot shows overall proteome turnover between phases and replicates for a single MAG. **d** Pearson correlation coefficients of transcriptome differences and proteome differences between phases for a single MAG (Supplementary Table 44). Each dot shows the correlation between the transcriptome and the proteome for a single MAG. Horizontal lines in (**b**–**d**) show significant differences determined with two-sample two-sided *t*-tests.

Metatranscriptomics and proteomics were used to determine changes to each population’s gene expression from the final oxic phase to the final anoxic phase (Fig. 5a and Supplementary Tables 35–42). Genes involved in all investigated metabolic categories, including the Calvin cycle, were active during both oxic and anoxic phases. At all three frequencies, genes for aerobic respiration were more actively transcribed during oxic phases, while denitrification genes were more active during anoxic phases. The responses of some subsystems were determined by the frequency of change. For example, hydrogen oxidation performed by NiFe hydrogenases was more active during the oxic phase at low-frequency and during the anoxic phase at high-frequency, respectively. Most subsystems showed no significant differences in proteomes.

To explore the role of gene expression in adaptation more broadly than these categories, we calculated the “turnover” of each population’s entire transcriptome and proteome across phases and frequencies. The turnover is the percentage of transcriptome/proteome that differs between two samples. To get a sense of the experimental noise and natural variability, we compared the turnover from the oxic to the anoxic phase of individual replicates to the turnover between two replicates at the same phase (oxic or anoxic).

Whereas transcriptome “turnover” between replicates was highest at high-frequency, transcriptome turnover between phases was highest at low-frequency (Fig. 5b and Supplementary Table 43). Exposure to change of higher frequency led to higher natural variability in transcriptomes. Only at low-frequency was transcriptome turnover associated with regulation (mean = 44.9%, SD = 15.4%, *n* = 63) higher than stochastic differences between replicates (mean = 24.0%, SD = 13.4%, *n* = 118) (two-sample two-sided *t*-tests; *p* = 2.5 × 10^−17^). Transcriptome turnover was always equal to or lower than cell turnover (two-sample two-sided *t*-tests; *p* ≤ 0.068), indicating that microbes may not have actively degraded old transcripts (messenger RNA), but only responded to change by adding new ones.

Surprisingly, proteomes showed a different trend (Fig. 5c and Supplementary Table 43): Here, turnover between replicates and phases were both highest at high-frequency. Proteome turnover between phases was similar to transcriptome turnover between phases at low-frequency (two-sample two-sided *t*-tests; *p* = 0.92), but much higher than transcriptome turnover between phases at high- (two-sample two-sided *t*-tests; *p* = 8.9 × 10^−29^) and medium-frequency (two-sample two-sided *t*-tests; *p* = 8.9 × 10^−10^). Proteome turnover was lower than cell turnover at medium- (two-sample two-sided *t*-tests; *p* = 9.3 × 10^−6^) and low-frequency (two-sample two-sided *t*-tests; *p* = 1.0 × 10^−47^), but larger than cell turnover at high-frequency (two-sample two-sided *t*-tests; *p* = 7.2 × 10^−27^). This indicated active proteome remodeling (degradation of old proteins). Alternatively, and perhaps more likely, these results could also be explained by active use of post-translational modifications (PTMs, see below) at high-frequency. Because many PTMs are unknown and not in our databases or might have been lost during sample processing, proteins with PTMs may not have been identified, artifactually increasing protein turnover numbers.

The coherence of transcriptional and translational responses was determined by calculating the Pearson correlation coefficient of mRNA and protein differences between phases (Fig. 5d and Supplementary Table 44). Significantly higher correlations were observed at low-frequency (mean = 0.26, SD = 0.36, *n* = 24) than high-frequency (mean = 0.0053, SD = 0.31, *n* = 50) (two-sample two-sided *t*-tests; *p* = 0.0023). Coherence between the transcriptome and proteome was thus only observed when the pace of change was lower than the generation time.

### Post-translational modifications

PTM is a mechanism for rapidly activating or suppressing a protein’s function. Phosphorylation and acetylation are two commonly observed PTMs [42]. In total, we observed 2320 phosphorylation events and 2003 acetylation events with high confidence across all 18 replicates and conditions (Fig. S6 and Supplementary Tables 45 and 46). We observed more phosphorylated proteins at medium-frequency (mean = 0.88% of detected proteins, SD = 0.49%) compared to the other two frequencies (mean = 0.28% of detected proteins, SD = 0.21%) (two-sample two-sided *t*-tests; *p* = 0.0034). Phosphorylation was mainly observed for enzymes involved in central metabolism (the TCA cycle and glycolysis), such as Enolase, Malate dehydrogenase and Phosphoglycerate kinase. Acetylated proteins were detected in similar amounts at the three frequencies (mean = 0.45% of detected proteins, SD = 0.18%) and were more often observed for proteins associated with the cell envelope, including membrane proteins, flagellar biogenesis, and regulators, such as molecular sensors and two-component response regulators.

## Discussion

In this study, we sampled a microbial community from a sulfidic spring, a community that was naturally exposed to redox gradients in space and time and was easily accessible year-round, facilitating future reproduction of the work. Next, we pre-adapted this community to the laboratory using weekly alternating oxic/anoxic conditions for 16 weeks. The pre-adaptation yielded an enrichment culture featuring at least 199 different populations, including many that were relatively abundant in the original spring. Although it remains unknown if a different frequency during the pre-incubation would have retained different populations, at least many remained at the start of the main experiment.

In the main experiment, the pre-adapted community was incubated at three different frequencies of oxic/anoxic change in three sets of triplicated chemostats. All chemostats had the same dilution rate, enforcing the same growth rate in all experiments. Though the total incubation time (and # of generations) at each frequency was different (Fig. 1), stabilization of community composition as well as concentrations of substrates and products indicated a pseudo steady state was reached long before the end of each experiment (Figs. 2 and 3). Thus, although this difference could have been a confounding factor, this did not appear to be the case in practice.

The amplicon sequencing data clearly showed at high-frequency, redox change selected for a single, generalist microbiome capable of coping with both oxic and anoxic conditions. In contrast, diverging aerobic and anaerobic microbiomes appeared at low-frequency. Even at low-frequency, populations displayed similar growth rates independent of redox conditions, as shown by co-expression of genes for both aerobic and anaerobic metabolism, similar peak-to-trough ratios in genome sequencing depth and persistent relative sequence abundances, with only few exceptions at low-frequency. Note that our low-frequency experiment featured 8-day long oxic and anoxic phases, much longer than common natural oscillations such as day/night cycles and feeding regimes. Thus, even though specialization of microbiomes was detectable, the enriched populations were overwhelmingly generalist. At an even slower pace of change, selection of specialized microbiomes will proceed eventually, as reported in previous studies, for example, seasonal change [43, 44].

We also investigated the use of transcriptional regulation and PTMs. We did not see an evident trend of PTMs at different frequencies, which might be explained by losses of PTMs during sample processing in combination with occurrence of an untested diversity in potential PTMs. Dedicated approaches to quantify PTMs will be needed to address the importance of PTMs more conclusively [45, 46]. Significant regulation was observed for some metabolisms on RNA level. For example, expression of genes involved in denitrification was higher during anoxic conditions, independent of frequency. In addition, the overall gene expressions were more variable at high-frequency. This variability was not associated with changing conditions but was stochastic, caused by differences between replicates.

In contrast, at low-frequency, variability in gene transcription could be mainly explained by redox state. This was also the only frequency at which transcriptomes and proteomes were overall consistent with each other. The discrepancy between transcriptional and translational regulation was observed previously. For example, abundance oscillations of proteins involved in central metabolisms of the cyanobacterium *Prochlorococcus* experiencing a 2-h light-dark cycle were substantially damped compared to the corresponding transcripts, while proteins and transcripts for some genes were completely antiphase [47]. Also in carbon-starved *Caulobacter crescentus* some genes reacted either on the mRNA or protein level, while other genes displayed opposite mRNA and protein responses [48]. Transcriptional regulation is commonly assumed to be a fast response, because a mRNA response is often observed immediately [49]. Whereas our study also detected rapid transcriptomic response for some genes, the entire transcriptome and proteome only aligned completely after multiple generations. It seems that transcriptional regulation could not be finished within a single generation. This was also reflected in some previous studies: for example, the transcriptomes of *Dinoroseobacter shibae* and *Candidatus Accumulibacter phosphatis* were still changing in response to oxic/anoxic change after more than half a generation [50, 51]. Other studies found transcriptomes to be significantly different multiple generations after a redox change [52, 53]. Transcriptomes are often used to probe microbial ecology and have been shown to have short (<10 min) half lives [54]. The much slower apparent response times observed here and in the cited studies might be important to consider in the design of future studies of ecosystems experiencing rapid change, such as intestinal [55], soil [56] and ocean-surface [57] microbiomes.

We compared the strength of the codon usage bias at different frequencies. Codon usage bias was previously associated with maximum growth rate (copiotrophs versus oligotrophs) [40]. In our experiments, success was not determined by differences in growth rate, as the growth rate was the same across all experiments. This was also observed in permeable sandy sediments, where oligotrophs were more abundant in more stable deeper sediments [41]. Because strong codon bias could enable copiotrophs to more rapidly respond to change, selection of copiotrophs at high-frequency can still be infered.

In conclusion, we incubated a sulfidic stream microbiome in replicated chemostats subjected to oxic/anoxic change at different frequencies. We found that generalists capable of both oxic and anoxic metabolism were more abundant than specialists and these microbes co-expressed genes for aerobic and anaerobic metabolisms continuously, independent of redox state. Individual populations expressed proteomes that enabled them to be active all the time, while transcriptional and translational regulation was only consistent at multi-generational timescales. Selection by high and low frequency of change was found to act on codon usage bias and this defines a novel perspective on this global feature of bacterial genomes. Future studies with different approaches and source microbiomes will validate if these findings can be generalized.

### Supplementary information

Supplementary Tables

Supporting Information

## Data Availability

All sequences of this study, including amplicons, metagenomes, metagenome-assembled-genomes and transcriptomes, are under the Bioproject PRJNA749639 (NCBI). The Biosamples of the 16S rRNA gene sequence are: SAMN20427124-SAMN20427234, SAMN26746688-SAMN26746699. The Biosamples for the metagenome raw reads are SAMN20395938-SAMN20395955 and the Biosamples for the metatranscriptome raw reads are SAMN20446884-SAMN20446901. The Biosamples for the MAGs are SAMN20395959-SAMN20395984. The mass spectrometry proteomics data have been deposited to the ProteomeXchange Consortium via the PRIDE partner repository [58] with the dataset identifier PXD028583.
