# Supplementary material for: Frequency of change determines effectiveness of microbial response strategies
Source: ISME J. 2023 Sep 18;17(11):2047–57. doi: 10.1038/s41396-023-01515-9 (PMC10579261; doi:10.1038/s41396-023-01515-9)
Supplement: Supplementary file 2 — Supporting Information [file 41396_2023_1515_MOESM2_ESM.docx]

Supplementary information for

**Frequency of change determines effectiveness of**

**microbial response strategies**

Shengjie Li^1,2,3^, Damon Mosier^1^, Xiaoli Dong^1^, Angela Kouris^1^, Guodong Ji^2^, Marc Strous^1^, Muhe Diao^1^*

^1^Department of Geoscience, University of Calgary, Calgary, AB T2N 1N4, Canada

^2^Key Laboratory of Water and Sediment Sciences, Ministry of Education, Department of Environmental Engineering, Peking University, Beijing 100871, China

^3^Department of Biogeochemistry, Max Planck Institute for Marine Microbiology, Bremen, 28359, Germany

*Corresponding author: Muhe Diao

Email address: muhe.diao@ucalgary.ca


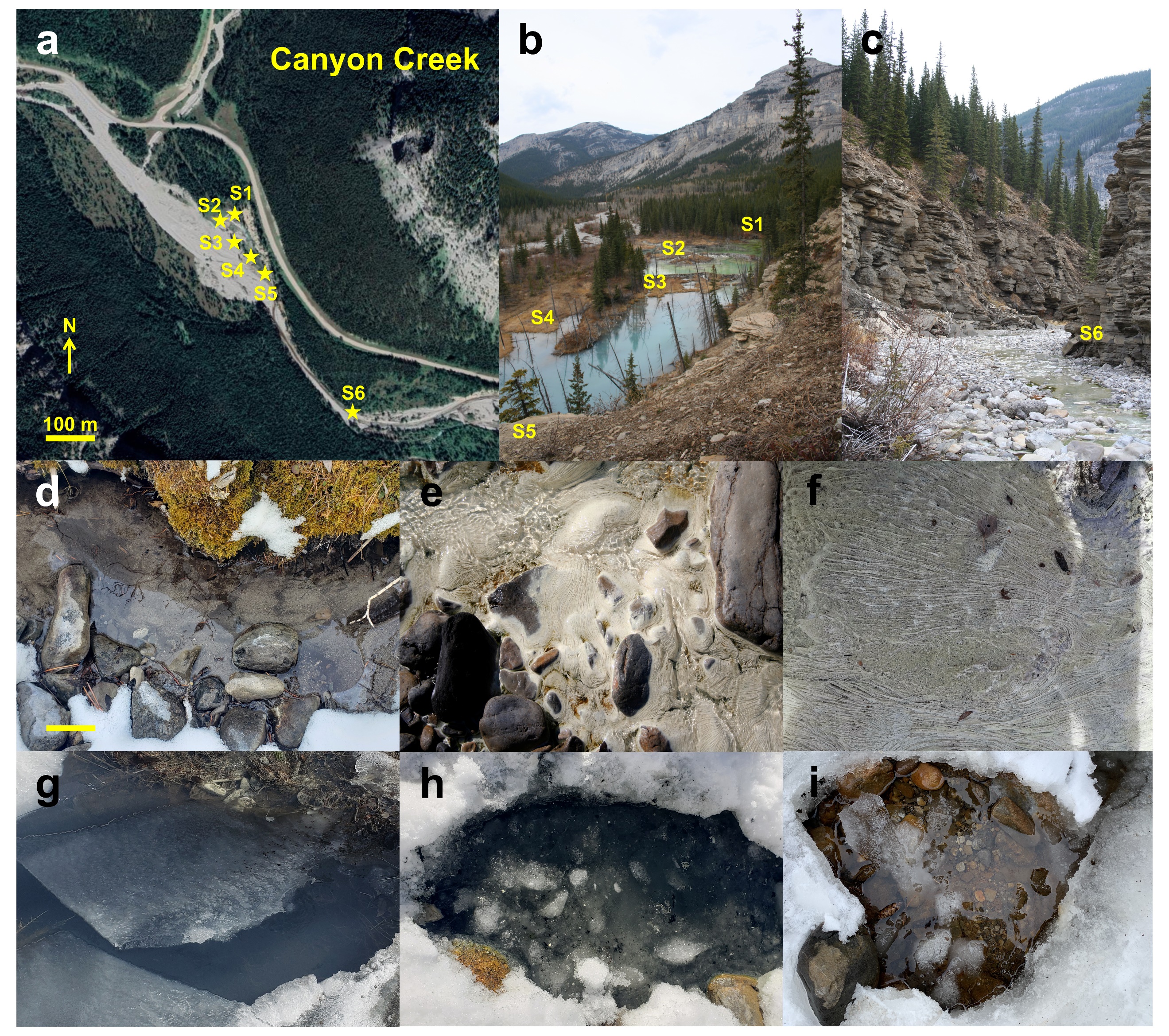


**Fig. S1 Sampling area and sites in Canyon Creek. a** Map of the sampling sites at Canyon Creek. The six sampling sites (S1-S6) were marked with yellow pentagrams. Map: Google Earth. **b** Photo of the locations and surroundings of S1 to S5. **c** Photo of the location and surroundings of S6. **d-i** Photos of in-situ conditions of S1-S6.


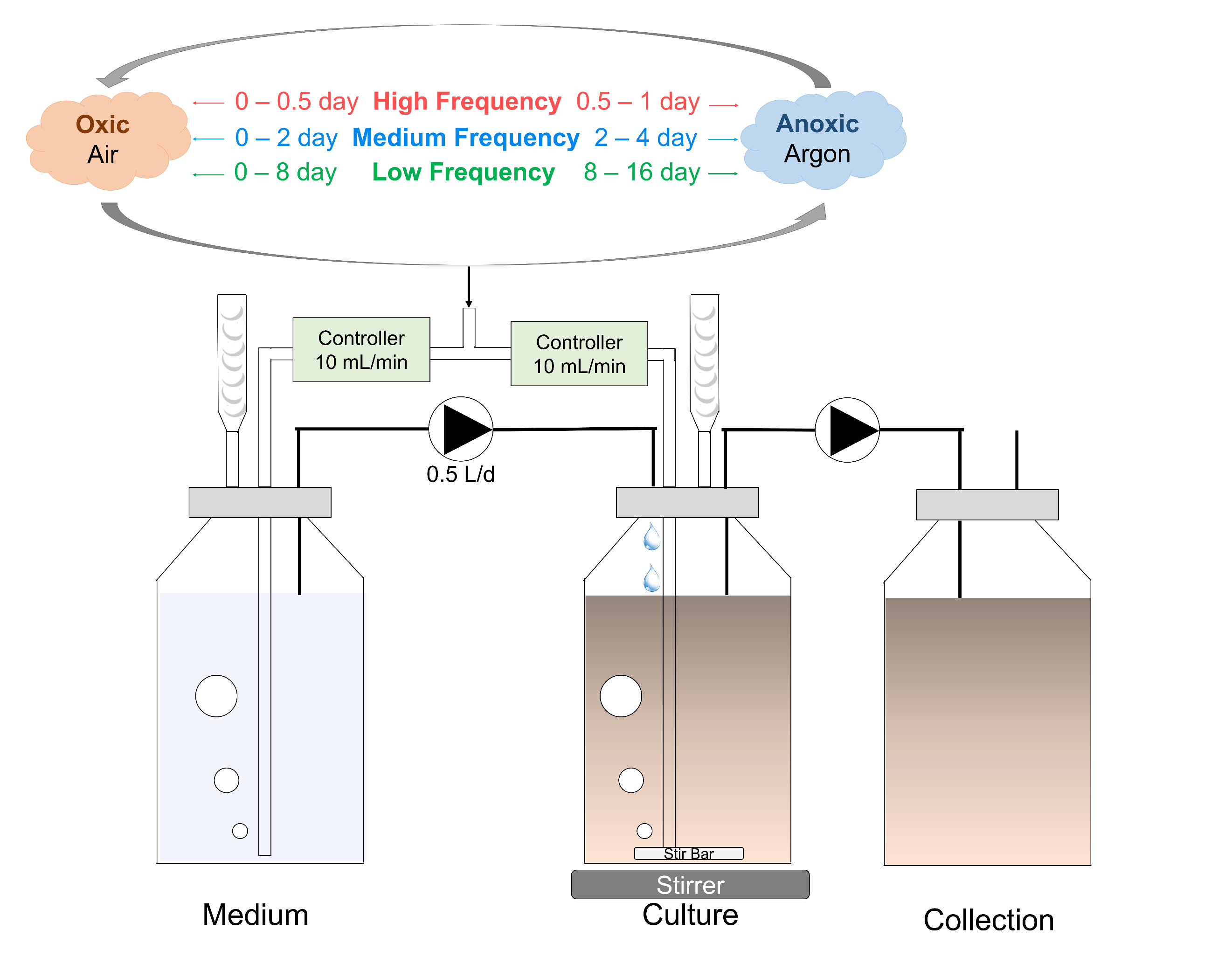


**Fig. S2 Experimental design of chemostats.** The chemostat setup consisted of a medium bottle, a magnetically stirred bottle and an effluent collection bottle. Fresh medium was pumped from the medium bottle to the culture bottle at a rate of 0.5 L per day. The total culture volume of the culture bottles was maintained by pumping out the excess culture volume to the effluent collection bottle. The chemostats experienced alternatingly oxic and anoxic conditions. Air was supplied to the medium bottle and the culture bottle during oxic phases, whereas Argon was supplied to the medium bottle and the culture bottle during anoxic phases. For high-frequency experiments, each phase lasted for 0.5 days. For medium-frequency experiments, each phase lasted for 2 days. For low-frequency experiments, each phase lasted for 8 days.


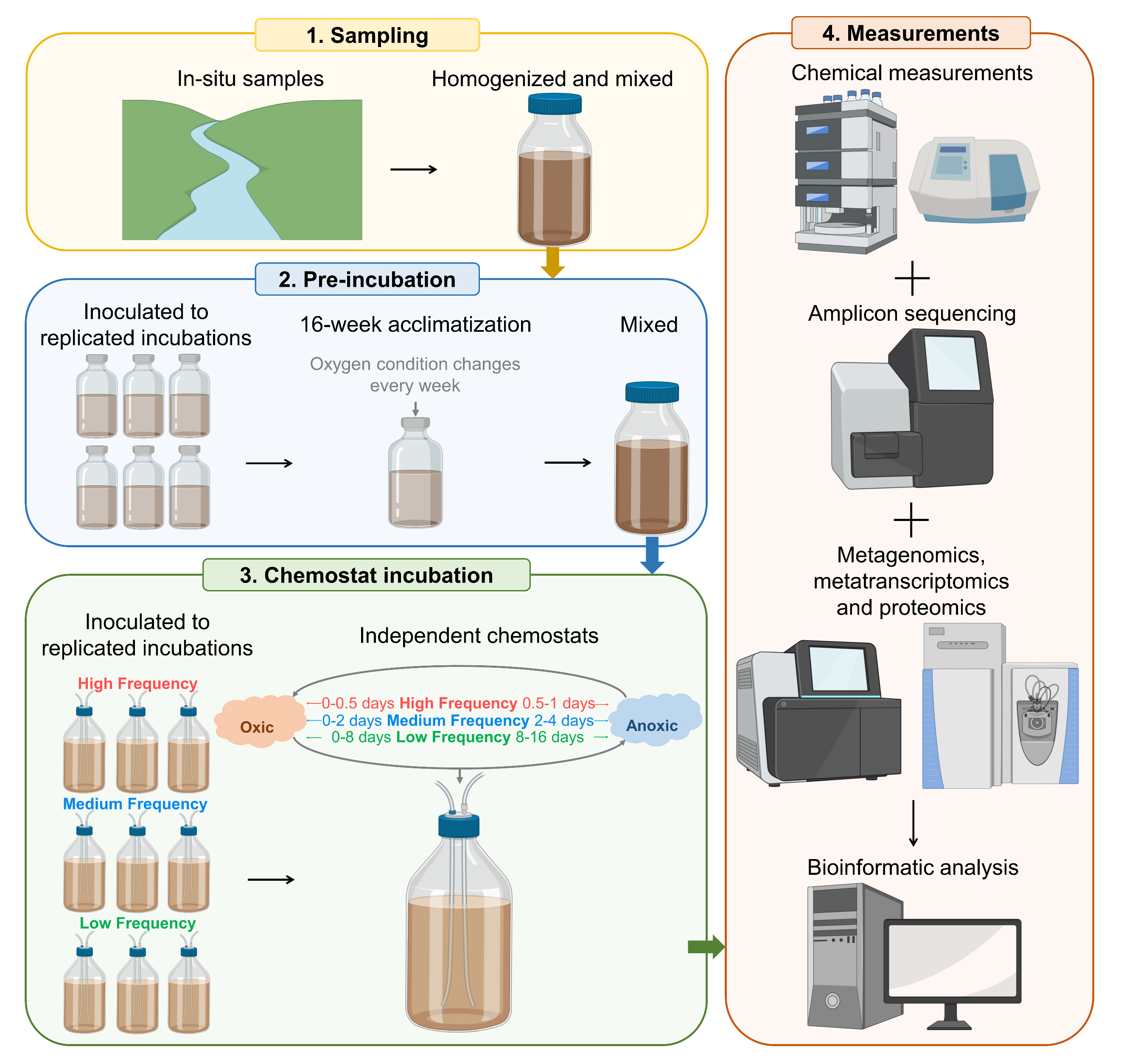


**Fig. S3 Workflow of the experiment.** Sediment samples were collected and homogenized. They were inoculated to six serum bottles and incubated for 16 weeks. During this period, the bottles were alternately incubated with and without oxygen for one week. Then the acclimatized cultures were mixed together and inoculated to nine chemostats. The chemostats experienced oxic and anoxic phases at different frequencies. Samples were collected from the chemostats and used for chemical measurements, amplicon sequencing, metagenomic sequencing, metatranscriptomic sequencing and metaproteomic measurements. Finally, bioinformatic analysis was conducted with the data.

**
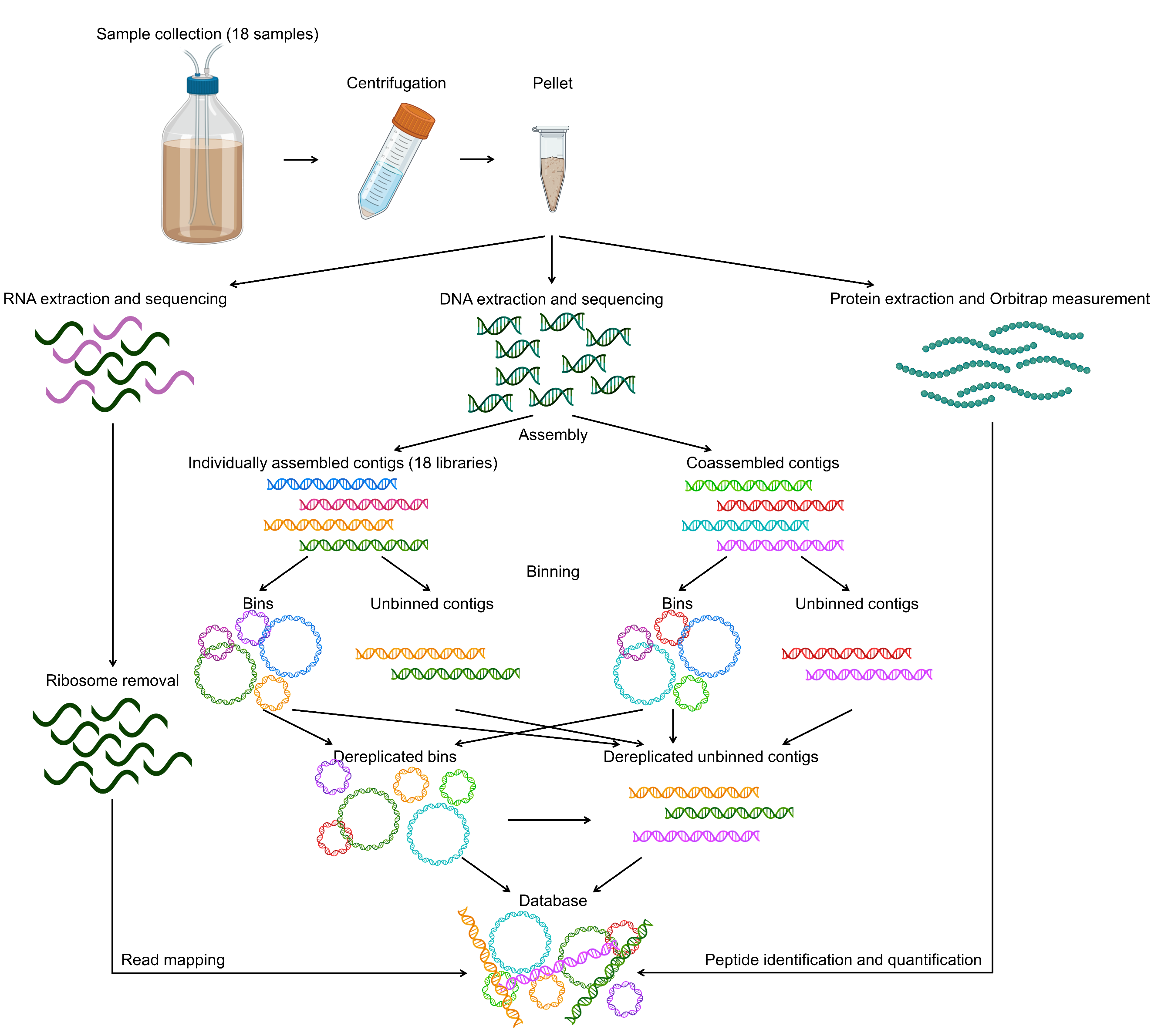
**

**Fig. S4 Workflow of multi-omic analysis.** Culture samples were collected from each chemostat. The samples were centrifuged and cell pellets were used for DNA, RNA and protein extractions. DNA reads were assembled separately for each sample and co-assembled for all samples. The coassembly and each individually assembled sample were binned separately. Bins and unbinned contigs from different assemblies were dereplicated and they were used as the metagenome database. RNA reads were mapped to the database after rRNA removal. Peptides were also identified and quantified based on the database.


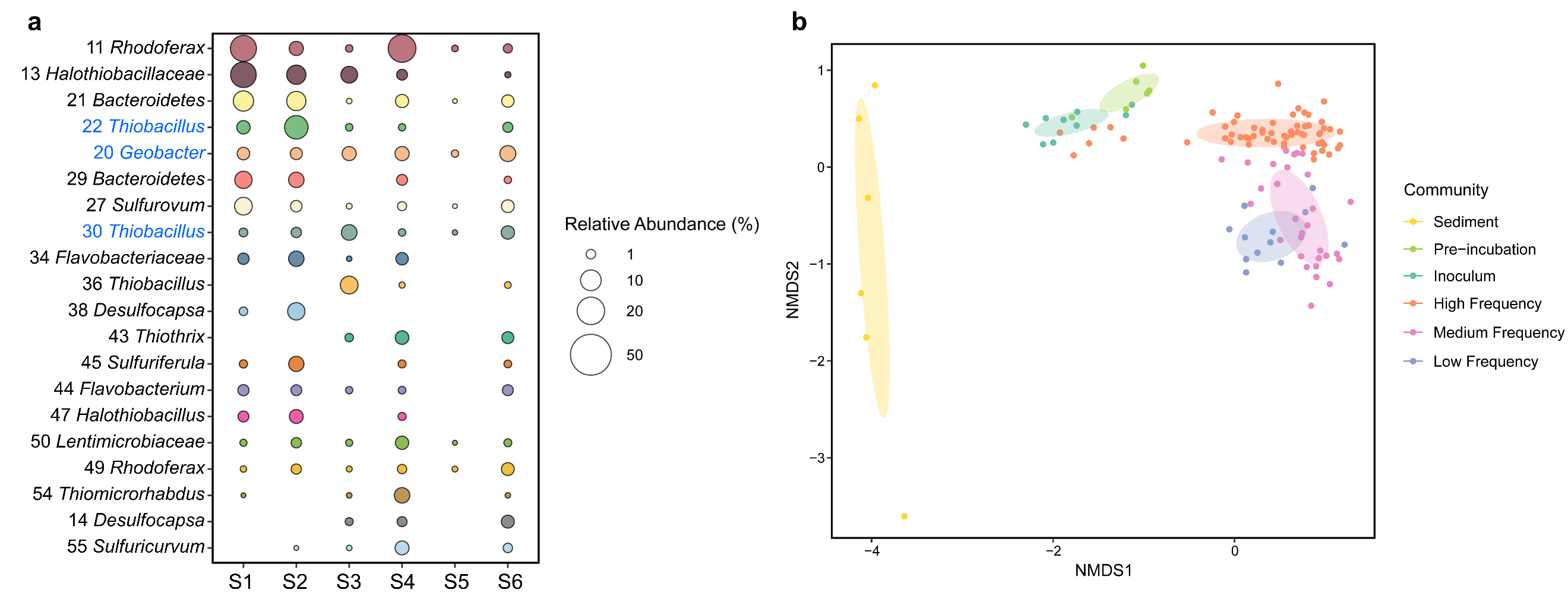


**Fig. S5 Microbial communities of in-situ sediments and enrichment cultures. a** Relative sequence abundance of the twenty most abundant populations (each associated with an amplicon sequence variant) in sulfidic stream sediments samples S1-S6 (Supplementary Table 2). The ASVs in blue color were represented in the chemostat incubations. **b** NMDS (based on Bray-Curtis distances) of in-situ sediment samples, pre-incubated samples in batch culture, inoculum samples for chemostats and all samples collected along the chemostat incubations. Samples were grouped using the ‘ordiellipse’ function from the ‘vegan’ package in R.


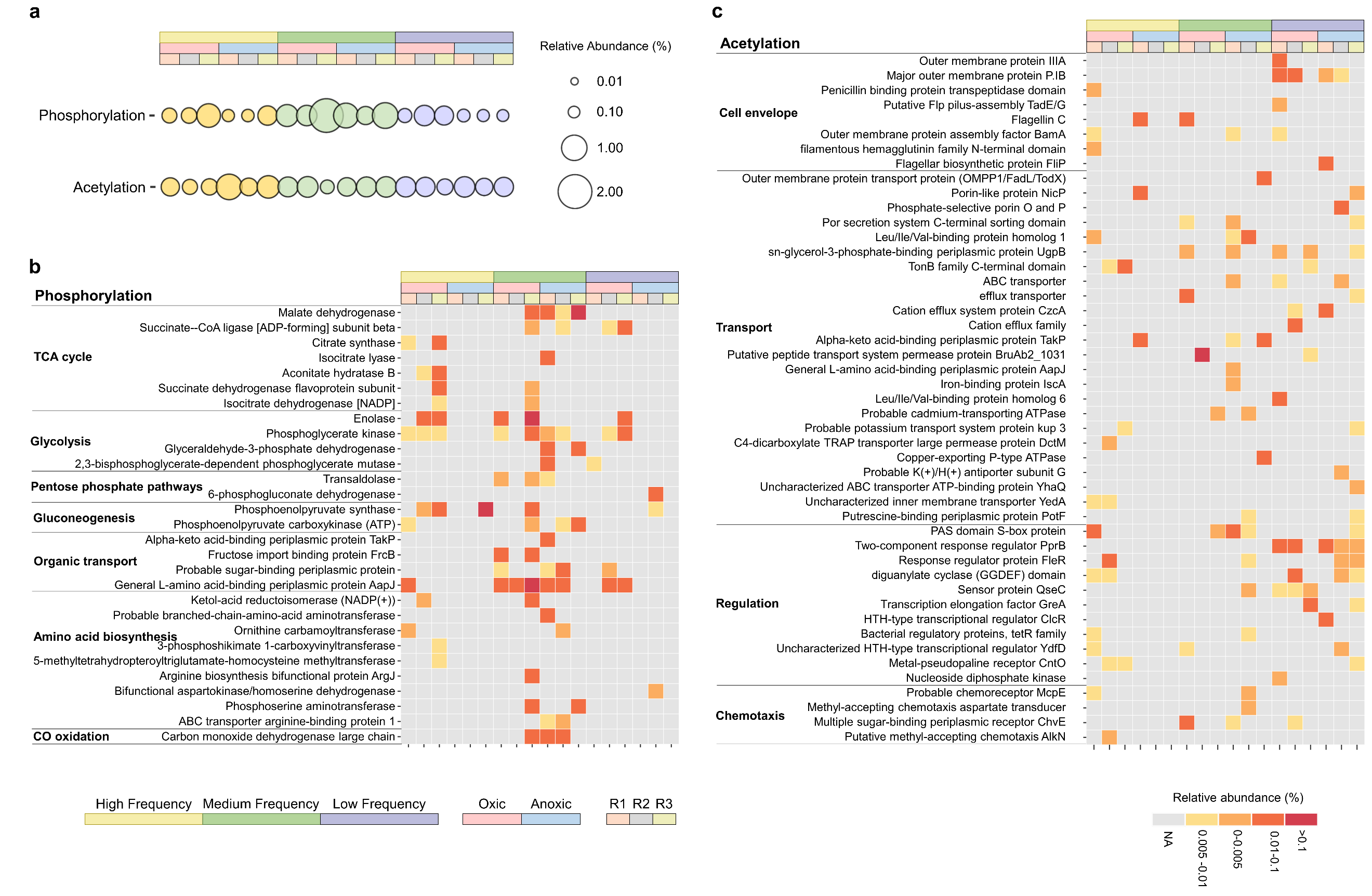


**Fig. S6 Post-translational modification events. a** Relative abundance of total phosphorylated and acetylated proteins (Supplementary Table 44). **b** Category and relative abundances of phosphorylated proteins. **c** Category and relative abundances of acetylated proteins (Supplementary Table 45).

**Supplementary Method**

In a chemostat, the mass balance of organisms is:

$\left[ Rate of accumulation of cells \right]=\left[ Rate of cells entering \right]-\left[ Rate of cells leaving \right]+[Rate of generation of live cells]$ (1)

In our design, $\left[ Rate of cells entering \right]$equals to zero because the medium is sterilized.

If we assume cells are not growing in the chemostat, $[Rate of generation of live cells]$equals to zero, and cell mass balance is worked as the followings:

$\left[ Rate of accumulation of cells \right]=V\frac{dC}{dt}$ (2)

$\left[ Rate of cells leaving \right]=-vC$ (3)

$V\frac{dC}{dt}=-vC$ (4)

Where $V$ is the culture volume, $C$ is concentration of cells, $v$ is the flowing rate of the effluent, $t$ is time. This can be reorganized in the following equation:

$\frac{dC}{C}=-Ddt$ (5)

Where $D$ is the dilution rate of the chemostat. In our design, per culture volume (1L) changes every 2 days, so $D$ equals to 0.5. The general solution to equation (5) is the following equation:

$lnC=-\frac{1}{2}t+a$ (6)

Where $a$ is a constant. At the beginning of the incubation, $t$ equals to zero and the cell concentration $\text{C}_{\text{o}}$ equals to $e^{a}$. At any given time, the cell concentration $\text{C}_{\text{t}}$ could be calculated as followings:

$\text{C}_{\text{t}}=e^{-\frac{t}{2}+a}=\text{C}_{\text{o}}e^{-\frac{t}{2}}$ (7)

$\frac{\text{C}_{\text{t}}}{\text{C}_{\text{o}}}=e^{-\frac{t}{2}}$ (8)

Assuming relative abundance of a population does not change between phases, theoretical percent of newly growing organisms of a population is calculated according to the following equation:

$Percent of newly growing organisms=100\%- percent of remained organisms$ (9)

$Percent of newly growing organisms=100\%-\frac{\text{C}_{\text{t}}}{\text{C}_{\text{o}}}$ (10)

$Percent of newly growing organisms=100\%-e^{-\frac{t}{2}}$ (11)

Where $\text{C}_{\text{t}}$ is the concentration of cells assuming cells are not growing in the chemostat. Equation (11) is obtained based on equation (8) and (10).

At high-frequency, when $t$ is to 0.5 days, the percent of newly growing organisms of a population is 22%.

At medium-frequency, when $t$ is 2 days, the percent of newly growing organisms of a population is 63%.

At low-frequency, when $t$ is 2 days, the percent of newly growing organisms of a population is 98%.
